# Harmonizing mind acupuncture combined with nicotine replacement therapy for tobacco use disorder: a protocol for a pragmatic randomized controlled trial

**DOI:** 10.3389/fpubh.2026.1850199

**Published:** 2026-07-16

**Authors:** Chuanxiong Li, Binyang Wang, Song Zhao, Qianwen Ruan, Lei Pan, Meihua Qiu

**Affiliations:** 1 The Affiliated Hospital of Yunnan University, Kunming, Yunnan, China; 2 Kunming Medical University, Kunming, Yunnan, China; 3 The Second Affiliated Hospital of Kunming Medical University, Kunming, Yunnan, China; 4 Second Affiliated Hospital of Yunnan University of Chinese Medicine, Kunming, Yunnan, China; 5 Yunnan University of Chinese Medicine, Kunming, Yunnan, China

**Keywords:** acupuncture, nicotine replacement therapy, pragmatic trial, randomized controlled trial, smoking cessation, tobacco use disorder, traditional Chinese medicine

## Abstract

**Background:**

Tobacco use disorder (TUD) remains a leading cause of preventable morbidity and mortality worldwide. Although nicotine replacement therapy (NRT) is recommended as a first-line treatment, its effectiveness is limited by persistent craving and withdrawal symptoms, resulting in high relapse rates. Acupuncture has been investigated as an adjunctive therapy; however, existing evidence is inconsistent and limited by methodological heterogeneity. High-quality pragmatic randomized controlled trials are needed. The co-primary outcomes are nicotine craving reduction (VAS) and biochemically verified 7-day point prevalence abstinence (PPA).

**Methods:**

This is a single-center, pragmatic, randomized, parallel-group controlled trial. A total of 220 participants with TUD will be randomly assigned (1,1) to receive either harmonizing mind acupuncture (HMA) plus NRT or NRT alone. The intervention period will last 6 weeks, with follow-up to 24 weeks. The co-primary outcomes are: (1) the change in nicotine craving measured by a visual analog scale (VAS) from baseline to week 6; and (2) 7-day point prevalence abstinence (PPA) biochemically verified by exhaled carbon monoxide at week 6. Secondary outcomes include exhaled carbon monoxide (as a continuous measure), quality of life, sleep quality, depressive symptoms, anxiety symptoms, and safety outcomes. Analyses will be conducted according to the intention-to-treat principle.

**Discussion:**

This pragmatic randomized controlled trial will evaluate the effectiveness and safety of HMA as an adjunct to NRT under real-world clinical conditions and provide clinically relevant evidence to inform integrative treatment strategies for TUD.

**Ethics approval and consent to participate:**

Ethical approval has been obtained from the Ethics Committee of the Affiliated Hospital of Yunnan University (Approval No.: KJR#20251111-R1-055). The study will be conducted in accordance with the Declaration of Helsinki. Written informed consent will be obtained from all participants prior to enrollment.

**Clinical trial registration:**

This trial has been registered with the International Traditional Medicine Clinical Trial Registry (ITMCTR) under registration number: ITMCTR2026000683 (https://itmctr.ccebtcm.org.cn/).

## 1 Introduction

Tobacco use disorder (TUD) represents a substantial global public health burden and is responsible for more than 8 million premature deaths annually, according to the World Health Organization (1). It is a major risk factor for cardiovascular disease, respiratory disorders, and multiple types of cancer. Despite extensive tobacco control policies and public health interventions, achieving sustained smoking abstinence remains challenging. The strong addictive properties of nicotine, together with a range of psychological and physiological withdrawal symptoms, contribute to high relapse rates (2–4).

TUD is characterized by compulsive tobacco use and frequent relapse, even among individuals aware of its harmful consequences (5). Common withdrawal symptoms—including craving, irritability, anxiety, depressed mood, and sleep disturbances—may persist for weeks or months and undermine cessation attempts (6). Effective treatment strategies therefore need to address both neurobiological dependence and behavioral regulation (7). Although nicotine replacement therapy (NRT), delivered via patches, gums, or lozenges, is widely used to reduce withdrawal symptoms, its effectiveness is limited by adverse effects and relapse after treatment discontinuation (8, 9). These limitations highlight the need for adjunctive interventions targeting both the physical and psychological aspects of nicotine dependence (9).

Acupuncture, a key modality of traditional Chinese medicine, has been investigated as a complementary approach for addiction treatment (10). Previous studies suggest that acupuncture may alleviate nicotine craving and withdrawal-related symptoms, potentially through modulation of neurochemical pathways and autonomic nervous system activity (11). However, existing evidence remains inconsistent, partly due to heterogeneity in acupuncture protocols and study design (12).

The present study introduces HMA, an integrative protocol designed to address both physical and emotional symptoms associated with nicotine withdrawal. HMA integrates traditional meridian theory with neurofunctional acupuncture point selection to target craving, emotional dysregulation, and autonomic imbalance. This pragmatic randomized controlled trial aims to evaluate the effectiveness and safety of HMA in combination with NRT in individuals with TUD under real-world clinical conditions. The primary objective is to evaluate the effect of HMA plus NRT on nicotine craving reduction; secondary outcomes assess abstinence rates and psychological measures. HMA employs a multidomain acupuncture point strategy intended to influence central neural processes, stabilize emotional responses, and support physiological regulation. This integrative approach is designed to address multiple components of nicotine withdrawal that may not be fully targeted by pharmacotherapy alone. Although evidence for acupuncture in substance dependence remains mixed, particularly for auricular approaches, emerging studies suggest that body- and scalp-based protocols targeting prefrontal–limbic circuitry may be associated with reductions in craving and improvements in affective symptoms. This pragmatic trial is expected to contribute to the existing evidence base by evaluating a standardized, symptom-oriented acupuncture protocol in combination with NRT in a clinically relevant setting.

## 2 Methods

### 2.1 Study design and setting

This study is a pragmatic, randomized, single-center, parallel-group clinical trial designed to evaluate the effectiveness and safety of HMA as an adjunct to NRT in individuals with TUD.

The study has been approved by the Ethics Committee of the Affiliated Hospital of Yunnan University (approval number: KJR#20251111-R1-055) and registered with the International Traditional Medicine Clinical Trial Registry (ITMCTR2026000683). The protocol was developed in accordance with the SPIRIT 2013 guidelines and the Standards for Reporting Interventions in Controlled Trials of Acupuncture (STRICTA) recommendations (13–15). Written informed consent will be obtained from all participants prior to enrollment.

**Figure 1:**
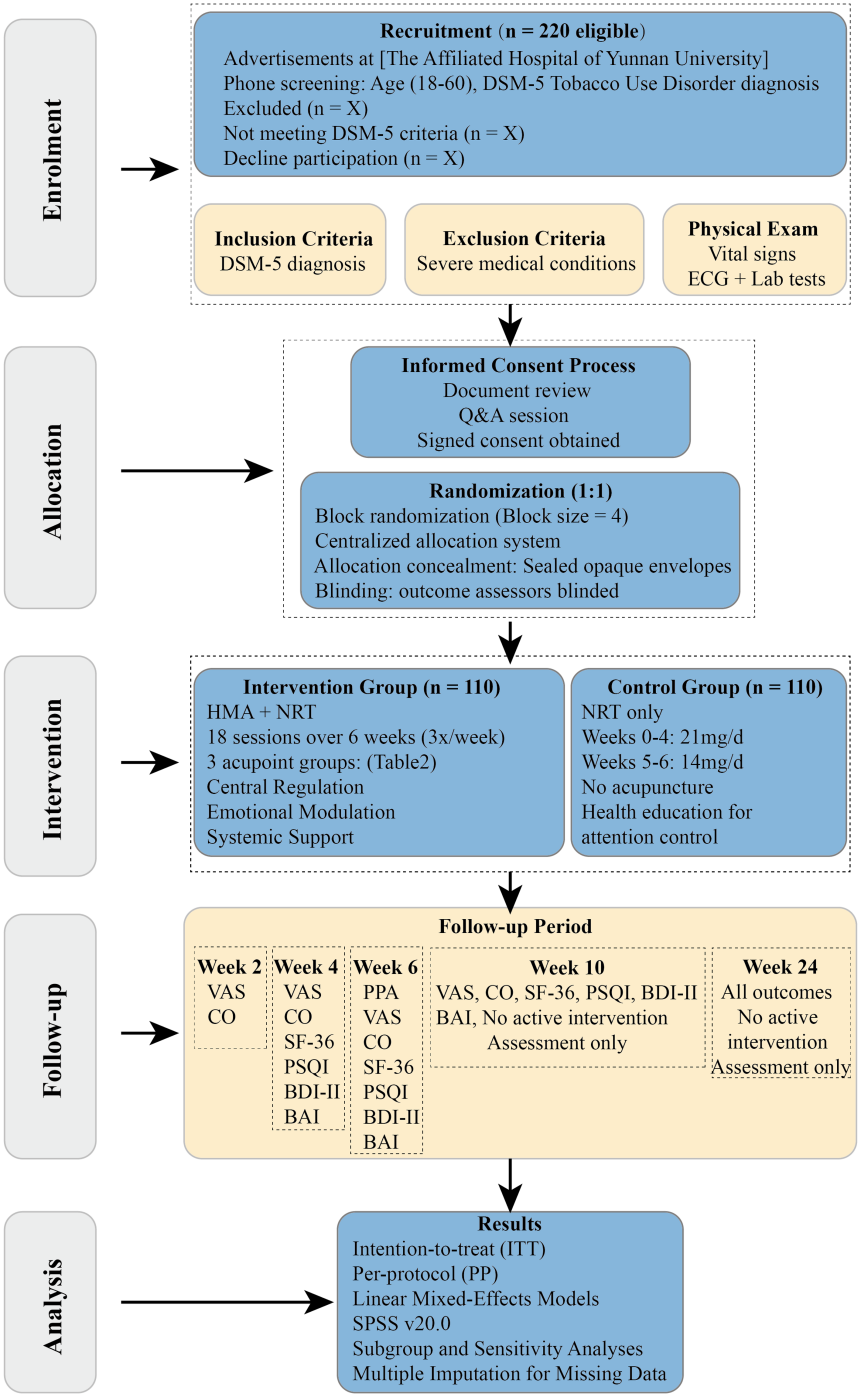
Flowchart of the pragmatic randomized controlled trial evaluating HMA combined with NRT. This diagram outlines the study design and procedural timeline, including screening, random allocation to two groups (HMA + NRT vs. NRT alone), the intervention schedule, assessment time points, and follow-up. The co-primary outcomes are: (1) change in nicotine craving intensity assessed by the visual analog scale (VAS) from baseline to week 6; and (2) 7-day point prevalence abstinence (PPA) at week 6 biochemically verified by exhaled carbon monoxide. Secondary outcomes include exhaled carbon monoxide (as a continuous measure), quality of life (SF-36), sleep quality (PSQI), depressive symptoms (BDI-II), and anxiety symptoms (BAI). Safety monitoring, including adverse event (AE) reporting and clinical evaluations, is conducted throughout the trial.

An overview of the trial flow is presented in Figure 1, and the schedule of enrollment, interventions, and assessments is shown in Table 1. This study is designed to assess the real-world effectiveness of HMA when used alongside standard nicotine replacement therapy in routine clinical practice.

**Table 1:** Participant flow and study timeline.

Timepoint (week)	-1	0	2	4	6	10	24
Enrollment phase							
Screening	×						
Informed Consent		●					
Allocation		●					
Intervention phase							
HMA + NRT Group		●	●	●	●		
NRT-only Group (+ health education for attention control)		●	●	●	●		
Assessment phase							
VAS (Nicotine Craving) (Co-primary outcome)		●	●	●	●	●	●
PPA (7-day Point Prevalence Abstinence) (Co-primary outcome)					●		●
CO (Exhaled Carbon Monoxide)		●	●	●	●	●	●
SF-36 (Quality of Life)		●		●	●	●	●
PSQI (Sleep Quality)		●		●	●	●	●
BDI-II (Depression Symptoms)		●		●	●	●	●
BAI (Anxiety Symptoms)		●		●	●	●	●
Safety monitoring							
Adverse events		●	●	●	●	●	●

This table presents a detailed overview of the study schedule, including participant enrollment, administration of interventions (HMA plus NRT or NRT alone), assessment timepoints for outcome measures, and safety monitoring (adverse events). Black dots (●) indicate the scheduled activities at each timepoint. The co-primary outcomes are: (1) change in VAS from baseline to week 6; and (2) 7-day point prevalence abstinence (PPA) at week 6.

### 2.2 Participants

#### 2.2.1 Recruitment and screening

Participants will be recruited through posters and institutional bulletins at the Affiliated Hospital of Yunnan University. Individuals who express interest will undergo an initial screening conducted by trained study personnel to assess eligibility based on predefined inclusion and exclusion criteria. Baseline and post-randomization treatment expectancy will be measured using the CEQ. Between-group differences will be incorporated into statistical models if imbalances are detected (16). Baseline assessments will include nicotine dependence severity (FTND), prior quit attempts, use of e-cigarettes or other nicotine products, and concurrent behavioral support (17).

#### 2.2.2 Inclusion criteria

Participants meeting all of the following criteria will be eligible for inclusion:

(1) Aged 18–60 years;
(2) Diagnosis of TUD according to the DSM-5 (18);
(3) Smoking at least 10 cigarettes per day for at least 1 year;
(4) Willingness to quit smoking and to initiate and adhere to nicotine replacement therapy as specified in the study protocol;
(5) No acupuncture treatment within the previous 3 months;
(6) Ability to provide written informed consent and comply with study procedures.

#### 2.2.3 Exclusion criteria

Participants meeting any of the following criteria will be excluded:

(1) History of severe cardiovascular, hepatic, renal, or pulmonary disease;
(2) Uncontrolled or severe comorbid conditions (e.g., decompensated liver cirrhosis, end-stage renal disease, unstable cardiovascular disease, active malignancy, or active tuberculosis);
(3) Severe or unstable gastrointestinal disorders (e.g., active inflammatory bowel disease or recent gastrointestinal bleeding) or clinical malnutrition;
(4) Severe or unstable psychiatric disorders (e.g., active psychosis, untreated major depressive disorder with severe symptoms, or bipolar disorder in a manic phase) or significant cognitive impairment affecting informed consent or protocol adherence;
(5) Use of concurrent medications or therapies that may confound study outcomes;
(6) Local skin infection, inflammation, scarring, or trauma at intended acupuncture sites;
(7) Pregnancy or intention to conceive during the study period.

### 2.3 Randomization, blinding and sample size

The use of a usual-care control group (NRT alone) rather than a sham acupuncture comparator reflects the pragmatic objective of this trial. The primary aim is to evaluate the incremental effectiveness of harmonizing mind acupuncture when added to standard smoking cessation therapy in real-world clinical settings.

Although sham acupuncture is commonly used in explanatory trials, its validity as a physiologically inert control remains debated.

Therefore, this study prioritizes clinical relevance and applicability by comparing HMA plus NRT with NRT alone, consistent with routine integrative practice.

Eligible participants will be randomly assigned in a 1:1 ratio to the intervention group (HMA plus NRT) or the control group (NRT alone) using a computer-generated block randomization scheme with a block size of four. The randomization sequence will be generated by an independent statistician using the REDCap platform (version 13.0; Vanderbilt University) and will be concealed from all study personnel involved in participant recruitment, intervention delivery, and outcome assessment (19).

Allocation concealment will be ensured using the sequentially numbered, opaque, sealed envelope (SNOSE) method (20). Envelopes will be opened sequentially by a designated study coordinator only after completion of baseline assessments.

Due to the nature of the acupuncture intervention, blinding of participants and acupuncturists is not feasible. However, outcome assessors, data managers, and statisticians will remain blinded to group allocation. Group assignments will be coded using anonymized labels (e.g., Group A and Group B) to minimize detection and analysis bias.

The sample size was calculated based on the co-primary outcome of 7-day point prevalence abstinence (PPA) at week 6. Referring to recent systematic reviews of acupuncture smoking cessation trials (21, 22), a control group abstinence rate of approximately 15% was assumed, with an odds ratio (OR) of 1.9 for the HMA + NRT group. With a two-sided significance level of *α* = 0.05, 80% power, and a 1:1 allocation ratio, a total of 188 participants is required. Accounting for a 15% dropout rate, the final sample size is set at 220 participants (110 per group) (23). The VAS craving outcome, as the other co-primary endpoint, is also adequately powered based on the same sample size. Multiplicity control. Because two co-primary outcomes are designated, the overall type I error rate is controlled using the Hochberg procedure. Under this approach, each outcome is tested at the full *α* = 0.05 level; the family-wise error rate is preserved because the trial is considered positive only if both co-primary outcomes achieve statistical significance (*p* ≤ 0.05 each). This method offers greater power than Bonferroni correction while maintaining strong control of the family-wise error rate.

To monitor expectancy and attention effects, participants will complete the Credibility/Expectancy Questionnaire (CEQ) at baseline and post-randomization; these scores will be explored in sensitivity analyses (16).

### 2.4 Interventions

Acupuncture interventions will be delivered by licensed acupuncturists with at least 3 years of clinical experience. The HMA protocol is based on classical meridian theory and adapted from prior randomized controlled trials, with refinement by a panel of senior experts. All practitioners will undergo standardized training before trial initiation, and a detailed procedural manual will be provided to ensure protocol adherence and minimize inter-operator variability.

**Figure 2:**
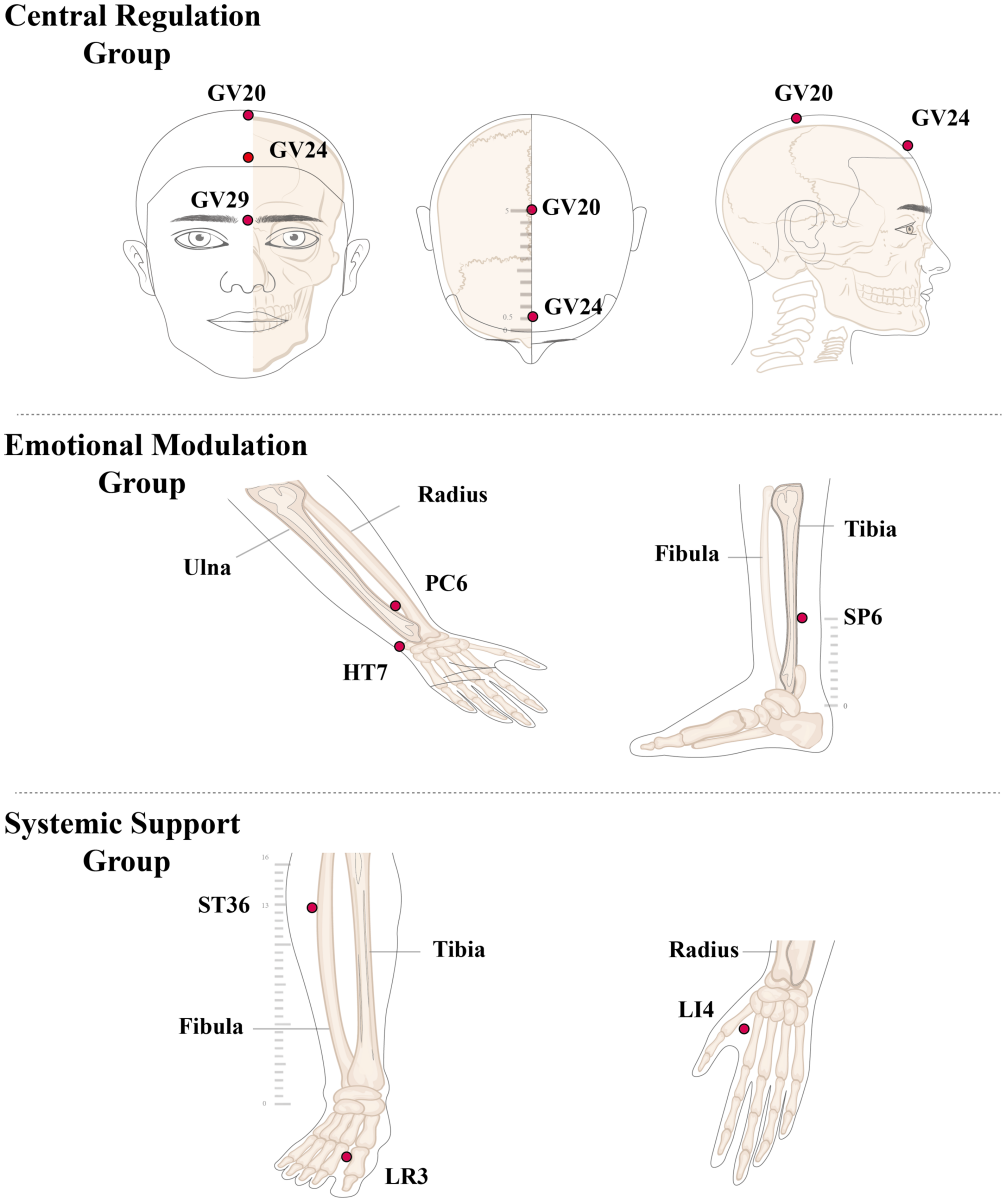
Anatomical Illustration of Acupuncture Points for the HMA Protocol. This diagram depicts the specific acupuncture points used in the HMA protocol, divided into three functional groups: Central Regulation (GV20, GV24, GV29), Emotional Modulation (PC6, HT7, SP6), and Systemic Support (ST36, LI4, LR3). These points target the neurological and psychological symptoms of TUD, aiming to regulate craving, emotional stability, and physical resilience during nicotine withdrawal.

Participants in the intervention group will receive HMA in addition to a standardized NRT regimen (identical to the control group). Acupuncture sessions will last approximately 30 min and will be administered three times per week for 6 weeks (total of 18 sessions). The control group will receive NRT alone. Following the treatment phase, participants will enter a follow-up period with assessments at Weeks 10 and 24. The intervention schedule is summarized in Table 1, with detailed acupuncture procedures provided in Tables 2, 3 and Figure 2. All participants will receive standardized brief behavioral counseling consistent with clinical smoking cessation guidelines (24).

**Table 2:** HMA Acupuncture point grouping and functional classification.

Group	Acupuncture point	Code	Description
Central regulation	Baihui	GV20	At the intersection of the midline of the head and the line connecting the apexes of both ears.
	Shenting	GV24	0.5 cun directly above the midpoint of the anterior hairline.
	Yintang	GV29 (EX-HN3, also referred to as GV29)	At the glabella, the midpoint between the medial ends of the two eyebrows, on the anterior midline of the head.
Emotional modulation	Neiguan	PC6	2 cun above the transverse crease of the wrist, between the tendons of palmaris longus and flexor carpi radialis.
	Shenmen	HT7	At the ulnar end of the transverse crease of the wrist, in the depression on the radial side of the tendon of flexor carpi ulnaris.
	Sanyinjiao	SP6	3 cun directly above the tip of the medial malleolus, posterior to the medial border of the tibia.
Systemic support	Zusanli	ST36	3 cun below ST35, one finger breadth from the anterior crest of the tibia, in the tibialis anterior muscle.
	Hegu	LI4	On the dorsum of the hand, between the 1st and 2nd metacarpal bones, at the midpoint of the 2nd metacarpal bone on the radial side.
	Taichong	LR3	On the dorsum of the foot, in the depression distal to the junction of the first and second metatarsal bones, approximately 1.5 cun proximal to the web margin between the big toe and second toe.

This table presents the acupuncture points applied in HMA, categorized into functional domains including central regulation, emotional modulation, and systemic support. Each point is defined by its anatomical location and therapeutic relevance in TCM. Acupoints PC6, HT7, SP6, ST36, LI4, and LR3 are applied bilaterally, whereas GV20, GV24, and GV29 are applied on the midline.

**Table 3:** Characteristics of De Qi sensations and needling parameters.

Acupuncture point	De Qi sensation	Needling sensation description	Needling depth	Needling angle	Remarks
GV20	Aching, Numbness, Heaviness	Local soreness and spreading heaviness	10–20 mm	15–30°	
GV24	Aching, Numbness, Heaviness	Mild soreness radiating to the forehead	10–20 mm	15–30°	
GV29	Aching, Numbness, Heaviness	Subtle pressure and light tingling	5–10 mm	15–30°	
PC6	Aching, Numbness, Heaviness	Tingling and radiating sensation toward the hand	10–25 mm	45–90°	
HT7	Aching, Numbness, Heaviness	Soreness with calming dull sensation	5–15 mm	45–90°	
SP6	Aching, Numbness, Heaviness	Deep aching and mild distension	15–30 mm	45–90°	
ST36	Aching, Numbness, Heaviness	Heavy sensation spreading to the lower limb	20–30 mm	45–90°	
LI4	Aching, Numbness, Heaviness	Intense distending sensation radiating to fingers	10–20 mm	45–90°	
LR3	Aching, Numbness, Heaviness	Pressing pain and spreading warmth	15–25 mm	45–90°	

This table outlines the De Qi sensation characteristics, needling techniques, and clinical grouping of acupuncture points used in HMA for treating tobacco use disorder. The points are categorized into Central Regulation, Emotional Modulation, and Systemic Support groups based on their TCM functions.

#### 2.4.1 Harmonizing mind acupuncture intervention

##### 2.4.1.1 Rationale for acupuncture point selection

The HMA protocol comprises three functional acupuncture point groups—Central Regulation, Emotional Modulation, and Systemic Support—designed to address the multidimensional features of TUD. Compared with conventional approaches focusing on auricular or single-meridian strategies, HMA integrates classical meridian theory with neurofunctional principles.

The Central Regulation group (GV20, GV24, and GV29) is used to regulate cognitive and emotional functions. The Emotional Modulation group (PC6, HT7, and SP6) targets anxiety, mood disturbances, and sleep-related symptoms. The Systemic Support group (ST36, LI4, and LR3) is intended to enhance physiological regulation and alleviate somatic discomfort. Together, these point groups aim to address craving, emotional dysregulation, and physical symptoms associated with nicotine withdrawal.

##### 2.4.1.2 Neurophysiological considerations

Emerging evidence suggests that acupuncture may modulate brain regions involved in addiction and emotional regulation, including the prefrontal cortex, amygdala, hippocampus, and nucleus accumbens (25, 26). These regions are implicated in craving, impulse control, and stress responses (27, 28). Acupuncture stimulation has also been associated with modulation of dopaminergic pathways and endogenous neurotransmitter release, which may contribute to the alleviation of withdrawal symptoms (29, 30).

##### 2.4.1.3 Needling technique and safety

Sterile, single-use stainless steel needles (0.25 mm × 25 mm or 0.25 mm × 40 mm) will be inserted to depths of 10–20 mm, depending on anatomical location. Insertion angles will range from 15°–30° for cranial points and 45°–90° for limb points. Manual stimulation will be applied to elicit the De Qi sensation. Needles will be retained for 30 min, with stimulation applied every 10 min.

All procedures will follow standardized safety protocols. In the event of adverse reactions, treatment will be discontinued and managed appropriately by the attending acupuncturist.

#### 2.4.2 Control intervention (nicotine replacement therapy)

Both groups will receive the same NRT regimen. Participants in the control group will receive NRT alone for 6 weeks.

The NRT regimen consists of transdermal nicotine patches administered at a fixed dose of 21 mg/day for the first 4 weeks, followed by 14 mg/day for weeks 5–6 (31). No dose adjustment based on VAS scores will be performed to avoid confounding feedback between the intervention and NRT dosage. All NRT doses will be recorded as administered and included as a covariate in sensitivity analyses.

#### 2.4.3 Adherence monitoring and data collection

Participants will attend six in-person visits (baseline and Weeks 2, 4, 6, 10, and 24). Each visit will include:

(1) Compliance assessment: NRT patch verification and diary review (VAS scores and adverse events).
(2) Outcome assessments (Table 1):

- CO and VAS: all visits.
- PPA: Weeks 6 and 24.
- SF-36, PSQI, BDI-II, and BAI: baseline and Weeks 4, 6, 10, and 24.

#### 2.4.4 Behavioral counseling and attention control

All participants will receive standardized brief behavioral counseling (5–10 min per visit) based on the 5A model (Ask, Advise, Assess, Assist, Arrange), delivered by a trained research nurse. To balance clinical contact time between groups, participants in the NRT-only group will receive an additional 5–10 min of structured health education at each visit, matching the duration of acupuncture sessions in the intervention group. Health education topics are standardized and include general wellness, lifestyle modification, stress management, and smoking-related health risks, delivered using a predefined educational pamphlet to ensure consistency (24).

### 2.5 Ethical considerations

To ensure participant welfare, individuals in the control group will be offered a complimentary six-week course of harmonizing mind acupuncture (HMA) after completion of the study, in accordance with Good Clinical Practice (GCP) guidelines (32).

### 2.6 Outcomes

#### 2.6.1 Co-primary outcomes

(1) Nicotine craving intensity will be assessed using a visual analog scale (VAS), consisting of a 100-mm horizontal line ranging from 0 (no craving) to 100 (maximum craving) (31). Participants will indicate their current level of craving by marking a point on the scale. The change in VAS score from baseline to week 6 serves as one of the co-primary outcomes.
(2) Seven-day point prevalence abstinence (PPA) will be assessed at week 6. Self-reported abstinence over the preceding 7 days will be biochemically verified using exhaled carbon monoxide (CO). Participants with discordant self-reported and biochemical results (i.e., self-reported abstinence but CO ≥ 6 ppm) will be classified as non-abstinent (33). PPA at week 6 serves as the second co-primary outcome. PPA at week 24 will be analyzed as a secondary outcome.

VAS craving was selected as a co-primary endpoint due to clinical relevance and feasibility, while biochemically verified PPA provides an objective measure of cessation, together addressing both subjective withdrawal and confirmed abstinence (33).

#### 2.6.2 Secondary outcomes

(1) Exhaled carbon monoxide (CO) as a continuous measure will be assessed at all visits to reflect recent smoking behavior (34).
(2) Point prevalence abstinence at week 24 will be assessed as a secondary time point using the same definition as the co-primary PPA outcome (self-report + CO < 6 ppm) (33, 35).
(3) Quality of life will be assessed using the validated Chinese version of the Short Form 36 Health Survey (SF-36), which includes eight domains of physical and mental health (36).
(4) Sleep quality will be assessed using the Chinese version of the Pittsburgh Sleep Quality Index (PSQI) (37).
(5) Depressive symptoms will be assessed using the Beck Depression Inventory-II (BDI-II) (38).
(6) Anxiety symptoms will be assessed using the Beck Anxiety Inventory (BAI) (39).

Assessment time points for all outcomes are summarized in Table 1.

### 2.7 Safety assessments and adverse events

#### 2.7.1 Safety parameters

Safety assessments will include hematological, hepatic, renal, inflammatory, and cardiac parameters. Hematological tests will include white blood cell count, hematocrit, hemoglobin, and platelet count. Liver function will be assessed using aspartate aminotransferase (AST), alanine aminotransferase (ALT), and gamma-glutamyl transferase (GGT). Values exceeding three times the upper limit of normal (ULN) will prompt clinical evaluation. Renal function will be assessed using serum creatinine and blood urea nitrogen (BUN). Inflammatory status will be evaluated using erythrocyte sedimentation rate (ESR). Cardiac safety will be assessed using a standard 12-lead electrocardiogram (ECG) at baseline and after treatment completion (40).

#### 2.7.2 Adverse events reporting

Adverse events (AEs) will be defined as any untoward medical occurrences during the study, regardless of their causal relationship with the intervention. All AEs will be recorded in case report forms (CRFs), including onset time, clinical features, severity (mild, moderate, or severe), duration, management, and outcome. Serious adverse events (SAEs), including death, life-threatening events, hospitalization or prolonged hospitalization, significant disability, or congenital anomalies, will be reported to the institutional ethics committee within 24 h. The committee will review each SAE and determine whether protocol modifications or trial discontinuation are required.

#### 2.7.3 Ongoing safety monitoring

Safety monitoring will be conducted throughout the study. Decisions regarding participant withdrawal for safety reasons will be made by the principal investigator in consultation with the research team. Safety data will be reviewed regularly by an independent data monitoring committee. A summary of AEs during the treatment phase will be presented in Table 4.

**Table 4:** Adverse events and side effects monitoring.

Date	Patient ID	Adverse event	Severity	Outcome	Duration	Action taken	Follow-up and remarks
							
							
							
							

Template for recording adverse events during the study; this table will be completed after trial initiation and during study conduct.

### 2.8 Quality control, data management, and monitoring

Before participant enrollment, all study personnel will undergo standardized training to ensure adherence to the study protocol and operational procedures. Acupuncture treatments will be delivered by licensed acupuncturists with at least 3 years of clinical experience. A detailed protocol handbook, including stepwise HMA procedures and standardized operating procedures (SOPs), will be provided to ensure consistency in intervention delivery (41).

Clinical data will be collected using predesigned case report forms (CRFs) and entered into a secure, password-protected electronic data management system. Data entry will be performed by trained personnel blinded to group allocation. Double data entry and independent verification will be conducted to ensure data accuracy and completeness. Real-time validation checks and regular audits will be performed by certified clinical research associates.

All original CRFs and source documents will be securely stored at the Affiliated Hospital of Yunnan University in accordance with Good Clinical Practice (GCP) standards. Oversight and monitoring will be conducted by the hospital’s Clinical Research Unit, which operates independently of the study investigators and funding sources. On-site monitoring visits will be conducted quarterly to assess protocol adherence. The Clinical Research Unit may suspend or terminate the study in the event of major protocol violations, safety concerns, or significant data inconsistencies.

### 2.9 Patient and public involvement

Patients and/or the public were not involved in the design, conduct, reporting, or dissemination of this research.

### 2.10 Statistical analysis

An independent statistician contributed to the development of the statistical analysis plan and the sample size calculation. This statistician, who will remain blinded to group allocation and not involved in study conduct, will perform all analyses according to the pre-specified plan. All analyses will be conducted according to the intention-to-treat (ITT) principle, including all randomized participants in their assigned groups.

#### 2.10.1 Two co-primary outcomes will be analyzed as follows

Multiplicity control for co-primary outcomes. Type I error is controlled at the family-wise level using the Hochberg procedure. The trial will be declared positive only if both co-primary outcomes (VAS craving change and PPA at week 6) achieve statistical significance at *p* ≤ 0.05.

The change in nicotine craving intensity measured by the VAS from baseline to week 6 (one of the two co-primary outcomes) will be analyzed using linear mixed-effects models including fixed effects for group, time, and group × time interaction, with participant-specific random intercepts to account for repeated measurements.

The PPA at week 6 (the second co-primary outcome) will be analyzed using generalized estimating equations (GEE) with a logit link function and an exchangeable correlation structure. Results will be reported as adjusted odds ratios (ORs) with 95% confidence intervals (CIs).

For smoking abstinence outcomes, missing smoking-status data will be treated as non-abstinent in the primary analysis. For sensitivity analysis, a pattern mixture model under missing-not-at-random (MNAR) assumptions will be applied, assuming a relapse rate of 80–90% among participants with missing follow-up data (42).

Secondary continuous outcomes will be analyzed using linear mixed-effects models, including fixed effects for group, time, and group × time interaction, and random intercepts to account for within-subject correlation. Models will be adjusted for baseline values where appropriate. SF-36 total scores and PSQI global scores will be analyzed directly, with similar models applied to BDI-II, BAI, and exhaled carbon monoxide (CO).

To control for type I error due to multiple comparisons across the secondary outcomes (BDI-II, BAI, SF-36, PSQI, CO), a false discovery rate (FDR) procedure will be applied. Adjusted *p*-values will be reported alongside unadjusted estimates, and findings will be interpreted with appropriate caution (43).

Prespecified subgroup analyses will include three-way interaction terms (subgroup × treatment × time) within mixed-effects models to explore potential effect modification by sex (male/female) and age (<40 vs. ≥40 years).

Sensitivity analyses will include a per-protocol (PP) analysis excluding participants with less than 80% adherence, defined as attendance at fewer than 14 of 18 acupuncture sessions or NRT use on fewer than 80% of study days.

Between-group differences in clinical contact and expectancy scores will be explored and adjusted for in sensitivity analyses to account for attention-related effects (16).

## 3 Discussion

To our knowledge, no previous randomized controlled trials have specifically evaluated HMA as an adjunct to NRT for smoking cessation in individuals with tobacco use disorder. The HMA protocol is informed by classical meridian theory and contemporary neurofunctional models of addiction, with a focus on craving regulation, emotional modulation, and cognitive control.

**Figure 3:**
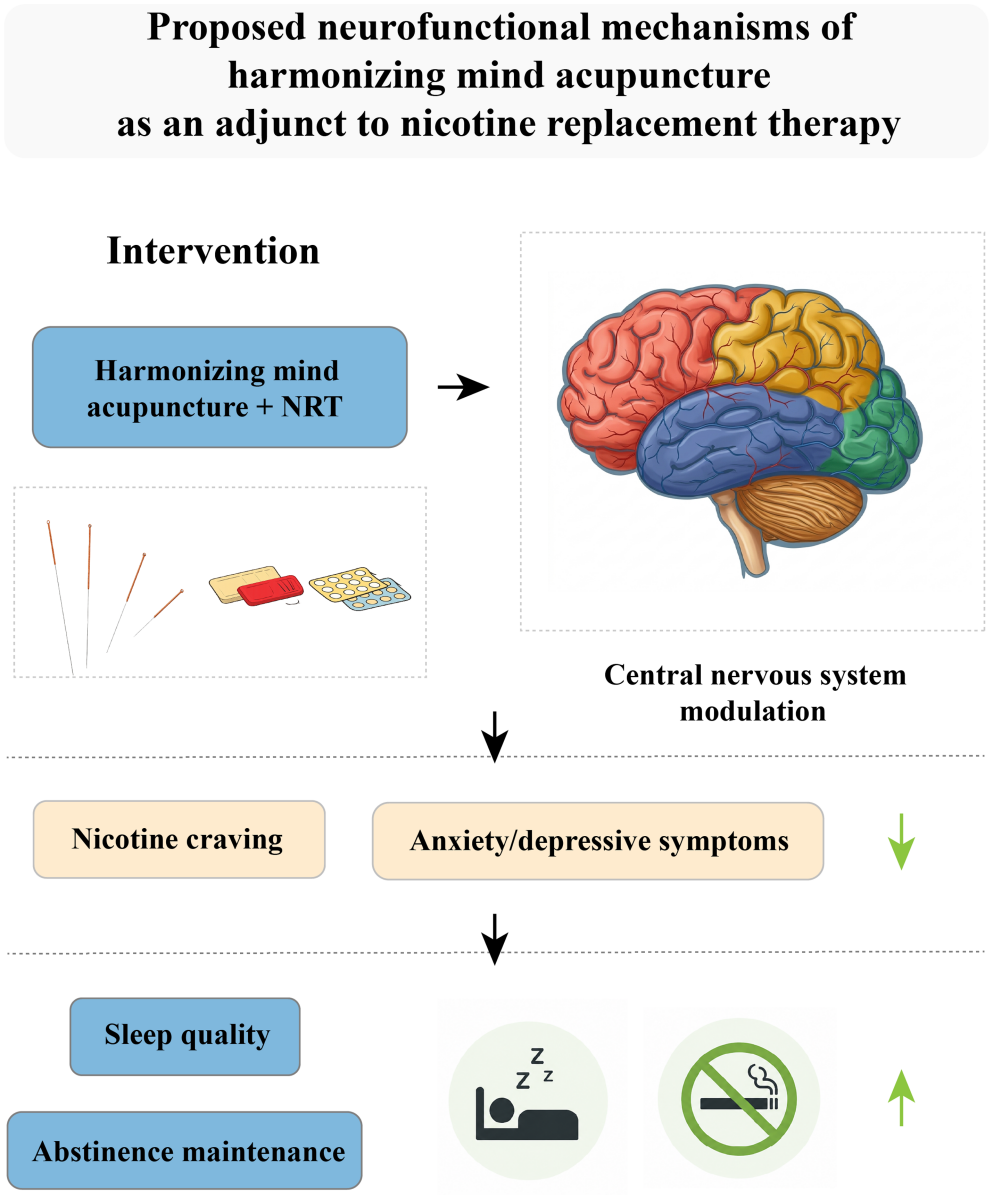
Proposed neurofunctional mechanisms of harmonizing mind acupuncture as an adjunct to nicotine replacement therapy. This schematic diagram illustrates a hypothesized neurofunctional pathway through which harmonizing mind acupuncture (HMA), when combined with nicotine replacement therapy (NRT), may influence smoking cessation outcomes. HMA is proposed to modulate central nervous system activity, particularly within the prefrontal cortex, amygdala, and related limbic structures involved in craving regulation, emotional processing, and stress response. Through these pathways, HMA may contribute to reductions in nicotine craving, anxiety, and sleep disturbance during withdrawal, thereby supporting abstinence maintenance. This figure represents a conceptual model based on existing neurobiological and acupuncture literature and is intended to be hypothesis-generating rather than evidence of confirmed biological mechanisms.

The proposed neurofunctional mechanisms underlying HMA are summarized in Figure 3 as a hypothesis-generating model. Detailed discussion of mechanisms is beyond the scope of this protocol.

This study incorporates several design features to enhance methodological rigor, including biochemical verification of smoking status using exhaled carbon monoxide, standardized practitioner training, and predefined data management and monitoring procedures. The findings may also inform the design of future trials incorporating sham controls and mechanistic endpoints. In particular, neuroimaging approaches may help clarify whether HMA influences neural circuits involved in craving, stress reactivity, and relapse vulnerability within prefrontal–limbic pathways, and may facilitate the identification of potential biomarkers of treatment response.

Further research is warranted to evaluate both short-term and long-term effects of combining HMA with NRT. While the present study focuses on withdrawal symptoms and cessation-related outcomes over a 24-week follow-up period, longer-term evaluation of relapse rates and sustained abstinence would provide additional evidence to inform clinical decision-making.

### 3.1 Limitations

This study has several limitations. First, the single-center design and relatively strict inclusion and exclusion criteria, including the exclusion of individuals with severe psychiatric comorbidities, may limit the generalizability of the findings to broader populations of smokers with tobacco use disorder.

Second, the absence of a sham acupuncture or attention-matched control limits the ability to distinguish specific effects of harmonizing mind acupuncture (HMA) from nonspecific or placebo-related effects, particularly for subjective outcomes such as visual analog scale (VAS) craving, BDI-II, and BAI scores. However, this design reflects the pragmatic objective of the trial to evaluate effectiveness in real-world clinical settings, and comparison with standard care (NRT alone) remains appropriate for informing clinical decision-making. To partially address this concern, biochemically verified 7-day point prevalence abstinence (PPA) has been included as a co-primary outcome, providing an objective measure of cessation that is less susceptible to expectancy effects. Differences in treatment exposure between groups may introduce attention-related bias, although baseline and post-randomization treatment expectancy scores will be collected and incorporated into sensitivity analyses to mitigate this effect.

Third, the 24-week follow-up period may be insufficient to fully capture long-term relapse patterns; future studies should consider extended follow-up durations of at least 52 weeks to better assess sustained abstinence and relapse risk.

Fourth, the technical complexity of the HMA protocol and the requirement to elicit De Qi sensations may introduce inter-practitioner variability despite standardized training and detailed procedural manuals.

Fifth, although missing smoking-status data will be handled as non-abstinent in the primary analysis, sensitivity analyses using MNAR assumptions may still be biased if dropout is related to unmeasured factors such as relapse or adverse events.

Sixth, while the sample size of 220 provides adequate power for both co-primary outcomes (VAS craving change and biochemically verified PPA), the single-center design and relatively strict inclusion criteria may still limit generalizability. Future multicenter studies with more diverse populations are warranted to confirm the findings.

Seventh, while this study focuses on the combination of HMA and NRT for craving reduction and smoking cessation, the mechanisms underlying acupuncture’s effects remain incompletely understood. Future studies incorporating sham controls, mechanistic endpoints, multicenter designs, and longer follow-up durations would strengthen causal inference and enhance the generalizability of findings.

## Glossary

AE: Adverse Event
ALT: Alanine Aminotransferase
AST: Aspartate Aminotransferase
BAI: Beck Anxiety Inventory
BDI-II: Beck Depression Inventory-II
BP: Bodily Pain
CRF: Case Report Form
PPA: 7-day Point Prevalence Abstinence
DSM-5: Diagnostic and Statistical Manual of Mental Disorders, Fifth Edition
ECG: Electrocardiogram
GABA: Gamma-Aminobutyric Acid
GH: General Health
GV: Governor Vessel (e.g., GV20, GV24, GV29)
HIV: Human Immunodeficiency Virus
HMA: Harmonizing Mind Acupuncture
HT: Heart Meridian (e.g., HT7)
ITT: Intention-to-Treat
LI: Large Intestine Meridian (e.g., LI4)
LR: Liver Meridian (e.g., LR3)
MH: Mental Health
NRT: Nicotine Replacement Therapy
PASS: Power Analysis and Sample Size software
PC: Pericardium Meridian (e.g., PC6)
PF: Physical Functioning
PP: Per-Protocol
PSQI: Pittsburgh Sleep Quality Index
RE: Role-Emotional
REDCap: Research Electronic Data Capture
RP: Role-Physical
SAE: Serious Adverse Event
SF: Social Functioning
SF-36: 36-Item Short Form Survey
SNOSE: Sequentially Numbered, Opaque, Sealed Envelopes
SP: Spleen Meridian (e.g., SP6)
SPIRIT: Standard Protocol Items: Recommendations for Interventional Trials
ST: Stomach Meridian (e.g., ST36)
STRICTA: Standards for Reporting Interventions in Controlled Trials of Acupuncture
TCM: Traditional Chinese Medicine
VAS: Visual Analogue Scale
VT: Vitality
TUD: Tobacco Use Disorder
CEQ: Credibility/Expectancy Questionnaire
