# Supplementary material for: Harmonizing mind acupuncture combined with nicotine replacement therapy for tobacco use disorder: a protocol for a pragmatic randomized controlled trial
Source: Front Public Health. 2026 Jul 16;14:1850199. doi: 10.3389/fpubh.2026.1850199 (PMC13422187; doi:10.3389/fpubh.2026.1850199)
Supplement: Supplementary file 1 [file Data_Sheet_1.docx]

# SPIRIT Checklist

Manuscript title:
Harmonizing Mind Acupuncture Combined with Nicotine Replacement Therapy for Tobacco Use Disorder: A Protocol for a Pragmatic Randomized Controlled Trial

| **SPIRIT Item** | **Section** | **Description** | **Reported information / Location** |
| --- | --- | --- | --- |
| Item 1 | Title | Identification of the study as a randomized controlled trial | Harmonizing Mind Acupuncture Combined with Nicotine Replacement Therapy for Tobacco Use Disorder: A Protocol for a Randomized Controlled Trial |
| Item 2 | Trial registration | Trial registration platform and identifier | Ethical approval has been obtained from the Ethics Committee of the Affiliated Hospital of Yunnan University (KJR#20251111-R1-055). The trial has been registered with the International Traditional Medicine Clinical Trial Registry (ITMCTR2026000683) |
| Item 3 | Protocol version | Date and version identifier | Protocol Version: YCYL. V.1.0, dated 15 July 2025 |
| Item 4 | Funding | Sources and types of financial support | Funded by Yunnan University Medical Research Foundation: YDYXJJ2024-0036, YDYXJJ2025-0037；Yunnan Fundamental Research Projects: 202501AT070739. |
| Item 5 | Roles and responsibilities | Names, affiliations, and roles of contributors | Author Contributions |
| Item 6 | Background and rationale | Scientific background and explanation of rationale | Introduction |
| Item 7 | Objectives | Specific objectives or hypotheses | Primary objective is to evaluate the efficacy and safety of harmonizing mind acupuncture as an adjunct to nicotine replacement therapy, with nicotine craving intensity assessed by visual analogue scale (VAS) as the primary outcome and smoking abstinence and other clinical measures as secondary outcomes. |
| Item 8 | Trial design | Description of trial design | single-center, parallel-arm, randomized controlled trial (1:1 allocation) |
| Item 9 | Study setting | Description of study settings | The Affiliated Hospital of Yunnan University |
| Item 10 | Eligibility criteria | Inclusion and exclusion criteria | Methods: Participants |
| Item 11 | Interventions | Description of interventions | Methods: Interventions; Tables 2–3 |
| Item 12 | Outcomes | Primary and secondary outcomes | Primary outcome: nicotine craving intensity assessed by VAS. Secondary outcomes: smoking abstinence (7-day PPA), exhaled CO, SF-36, PSQI, BDI-II, BAI, safety outcomes. |
| Item 13 | Participant timeline | Schedule of enrolment, interventions, and assessments | Table 1 |
| Item 14 | Sample size | How sample size was determined | Sample size was calculated based on the primary outcome (VAS), assuming 90% power, a two-sided alpha of 0.05, and an anticipated dropout rate of 15%. |
| Item 15 | Recruitment | Strategies for achieving adequate enrolment | Recruitment is expected to begin in June 2026. |
| Item 16 | Randomization | Sequence generation, allocation concealment, implementation | Methods: Randomization |
| Item 17 | Blinding | Who will be blinded | Methods: Blinding |
| Item 18 | Data collection methods | Plans for assessment and collection of outcome data | Methods: Data collection |
| Item 19 | Data management | Plans for data entry, coding, security, and storage | Methods: Data management |
| Item 20 | Statistical methods | Statistical methods for primary and secondary outcomes | Methods: Statistical Analysis |
| Item 21 | Monitoring | Data monitoring committee and harms | Methods: Safety monitoring |
| Item 22 | Ethics approval | Research ethics approval | Methods: Ethical Considerations |
| Item 23 | Consent | Informed consent procedures | Methods: Ethical Considerations |
| Item 24 | Confidentiality | How confidentiality will be maintained | Methods: Data management |
| Item 25 | Dissemination policy | Plans for dissemination of results | Methods: Dissemination |
| Item 26 | Patient and public involvement | Extent of patient and public involvement | Methods: Patient and Public Involvement |
